# REPLY

**Published:** 2004

**Authors:** Jaydip Sarkar

**Affiliations:** Consultant Forensic Psychiatrist, Shaftesbury Clinic, Springfield University Hospital, London SW17 7DJ, United Kingdom amdeep176@aol.com


					84
Sir,
The study of Sarkar and Chandra (2003) attempts to show
the prevalence of alexithymia and its association with
abnormal illness behaviour among a socioculturally
homogeneous group of patients with somatic symptoms. A
somewhat similar study (Radhika 1997) was conducted in
a general hospital psychiatry unit on 71 patients ( a rather
sociocultually heterogeneous but with 70% Hindu
population) presenting with somatic symptoms to the
medical and surgical departments and referred for
psychiatric help. The prevalence of alexithymia measured
by the Toronto Alexithymia Scale was rather low (7%)
among this population (mean score 58.53 +_ 10.95). The
results of one way analysis of variance on the score of
alexithymia among the diagnostic groups (anxiety,
depressive and somatoform disorder) showed that the score
of alexithymia was significantly higher among those with
somatoform disorder than among those with anxiety disorder
(p=0.001). A higher (a trend toward significance, p=0.077)
alexithymia scores were observed among the smaller
number of patients (N=15) fulfilling the criteria of abnormal
illness behaviour as per Illness Behaviour Assessment
Schedule than the other bigger group (N=56) who did not.
Again, a trend of significant positive correlation (r = 0.232,
p = 0.05) was found between the alexithymia scores and
the number of somatic symptoms present in an individual
patient in the entire study sample as well as among the two
gender groups separately. We also looked into the relation
between alexithymia characteristics and the range of coping
mechanisms of the patients. A non-significant negative
correlation was found only in the group of male patients.
Although such studies tend to indicate some kind of clinical
association of alexithymia, certain fundamental issues
remain to be addressed. Is the construct of alexithymia
cross culturally applicable? Is it possible to differentiate
between trait and state of alexithymia among the community
population? Does the presence of alexithymia tend to have
any clinical validity? Unless these issues are thoroughly
addressed and resolved in future research the relevance of
alexithymiatoourpopulationwillcontinuetoremainindoubt
in the sceptic mind of the clinician.
REFERENCES
Radhika C.R. (1997) : A study of alexithymia, illness behaviour and coping
repertoire among the patients with somatic symptoms, M.D. dissertation
submitted to Mangalore University.
Sarkar J. and Chandra P. (2003) : Alexithymia and illness behaviour among
female Indian outpatients with somatic symptoms, Indian Journal of
Psychiatry, Vol. 45, No. IV, 229-233.
Radhika C.R, Ex-post graduate student, Kasturba Medical
College, Manipal, Karnataka - 576 119.
Somnath Sengupta, Additional Professor of Psychiatry,
Institute of Human Behaviour and Allied Sciences
Dilshad garden, Delhi - 110 095.
REPLY
Sir,
Radhika and Sengupta raise many pertinent issues about
our study (Sarkar and Chandra, 2003) in their letter to the
editor. The discrepancy between the rates of alexithymia
between their and our study may be on account of several
factors, not least being the sociocultural heterogeneity of
their sample. It has been suggested that there are significant
differences between Hindu and Muslim patients who
present with multiples somatic symptoms, with Muslim
patients tending to have higher rates of unexplained somatic
complaints (Janakiramaiah and Subbakrishna, 1980). That
was the precise reason why we chose a socioculturally
homogenous sample. There is much evidence to suggest
that subjects from a non-English speaking background tend
to have higher scores on the Toronto Alexithymia Scale
(Le et al, 2002; Taylor et al, 2003) and the reasons for that
have been felt to be largely related to the cultural norms,
particularly to parental emotional socialisation, i.e. the
manner in which emotional expression is demonstrated by
parents to their children (Le et al, 2002).
The contribution of culture to the ability to experience
emotional distress, accept it, label it and then express it to
others cannot be underestimated especially since in eastern
(includingIndian)cultures,emotionaldistressisnotaccorded
the status of illness and is often felt to signify a personal
weakness of moral fibre in the individual expressing it,
thereby leading conversely to the use of body as an idiom
of distress. The construct of alexithymia has been
LETTERTOTHEEDITOR Indian Journal of Psychiatry, 2004, 46(I)84-85
Alexithymia and illness behaviour among female Indian outpatients
with somatic symptoms, Sarkar J. and Chandra P
.
Indian Journal of Psychiatry, Vol. 45, No. IV, 229-233.
85
examined and assessed in multiple cultures and the scale
translated into many languages, including Hindi (Pandey et
al, 1996), with adequate psychometric properties (Taylor
et al, 2003) to allow the conclusion, that it is an etic
(pancultural) as opposed to an emic (unicultural, culture-
bound) phenomenon (Taylor et al, 2003). It matters not
what one calls the phenomena but clinicians in India are
well aware that many of their patients complain in a
somatically focussed manner when the objective view may
be that the cause of their distress is psychological and the
use of the term alexithymia helps to label this phenomenon
and measure it.
Although there appears to be a wider recognition that
alexithymia is more likely to be a trait rather than state
phenomenon,thedebateisstillongoing. Longitudinalstudies
should answer such questions by assessing for the presence
of alexithymia over time, including but going beyond
episodes of somatic illness. Our study was not designed to
answer this question but our view is closer to that of
alexithymia being a trait phenomenon, with higher baseline
scores with further possible rises in scores during times of
somatic illnesses of non-organic nature.
Theauthorsaskwhetheralexithymiahasanyclinicalvalidity.
This appears to be a redundant question in the face of large
amount of literature that exists about the validity of this
construct. A more appropriate question may be whether it
has any clinical utility. Alexithymia has been found to be
associated with tonic physiological arousal, certain types
of unhealthy behaviour (e.g. substance misuse, eating
disorders), a biased perception and reporting of somatic
sensations and symptoms, and also appears to influence
excessive health care use, albeit in a complex manner
(Lumley, 1996). Presence of this trait in certain patients
should alert professionals to the possibility of over-reporting
of physical symptoms that may not be organically based,
thereby being of much use in the general medical setting.
It would also help target remedial measures such as
psychotherapy by helping the patient to establish links
between psychological distress and somatic symptoms. By
identifying such patients early, one can prevent unnecessary
investigations thereby allowing better utilization of, often
scant, existing resources. The advantages are therefore
many and are not merely restricted to direct benefits to the
patient and his doctor.
REFERENCES
Sarkar J., Chandra P (2003) Alexithymia and Illness Behaviour among
female Indian outpatients with multiple somatic symptoms. Indian Journal
of Psychiatry, 45 (IV), 229-233.
Janakiramaiah N., Subbakrishna D.K. (1980) Somatic neurosis in muslim
women in India. Social Psychiatry, 15, 203-206
Le H., Berenbaum H., Raghavan, C. (2002) Culture and Alexithymia:
Mean Levels, Correlates, and the Role of Parental Socialization of Emotions.
Emotion, 2 (4), 341-360.
Taylor G.J., Bagby R.M., Parker J.D.A. (2003) The 20-Item Toronto
Alexithymia Scale: IV. Reliability and factorial validity in different languages
and cultures. Journal of Psychosomatic Research, 55 (3), 277-283.
Pandey R., Mandal M.K., Taylor G.J., Parker J.D.A. (1996) Cross-cultural
alexithymia: development and validation of a Hindi translation of the
twenty-item Toronto Alexithymia Scale. Journal of Clinical Psychology
142, 1150-1155.
Lumley M.A., Stettner L., Wehmer F. (1996) How are Alexithymia and
physical illness linked? A review and critique of pathways. Journal of
Psychosomatic Research, 41(6), 505-518.
Jaydip Sarkar, Consultant Forensic Psychiatrist, Shaftesbury
Clinic, Springfield University Hospital, London SW17 7DJ,
amdeep176@aol.com
Indian Journal of Psychiatry, 2004, 46(I)85-86

				

